# High temperature performance of wire-arc additive manufactured Inconel 718

**DOI:** 10.1038/s41598-023-29026-9

**Published:** 2023-03-20

**Authors:** William Sean James, Supriyo Ganguly, Goncalo Pardal

**Affiliations:** Welding and Additive Manufacturing Centre, Cranfield University, Cranfield, MK43 0AL Bedfordshire UK

**Keywords:** Mechanical properties, Metals and alloys

## Abstract

In developing a wire-arc directed energy deposition process for superalloys used in high-speed flight environments, Inconel 718 was deposited using a plasma arc process and tested for its high temperature performance. The deposited material was tested in both the as deposited condition and after an age-hardening industry standard heat-treatment for this alloy. Results showed a reduced performance in both deposited conditions, with heat-treated material significantly outperforming as deposited material up to 538 °C. The difference in performance was less significant from 760 to 1000 °C, owing to an in-test aging process which increased the performance of the as deposited material. The microstructure of deposited material showed significant cracking throughout the alloy and formation of secondary phases throughout the matrix, with significantly more precipitation after heat-treating.

## Introduction

Cranfield University specialises in the development of directed energy deposition (DED) additive manufacturing (AM) processes. This study focuses on wire-arc DED, also known as Wire + Arc Additive Manufacturing (WAAM); where an electric arc is used to deposit a wire feedstock^1^, and where deposition rates are an order of magnitude greater than various other metal AM processes.

Many high-speed flight related applications require strength at high temperatures which necessitates the use of specialist alloys such as nickel-based superalloys, or Hastelloy. Production of these alloys using WAAM will allow significant cost reductions over conventional manufacturing by material saving and with greatly reduced lead times. In addition, it will greatly accelerate the development of new designs, as prototypes can be manufactured more quickly and more cost effectively. This paper will explore the effect the WAAM process has on the high temperature tensile properties of Inconel 718 (IN718).

Inconel 718 is an age hardened nickel-based superalloy, which is one of the most widely used alloys in aerospace engine components. IN718 was developed for high temperature service, as such it was designed for strength at higher temperature, creep resistance and good fatigue life up to 650 °C^2^.

An investigation into the room-temperature tensile properties and macrostructure of wire-arc DED IN718 has been investigated in a previous study by James et al., amongst other alloys. They found that as deposited (AD) age hardened alloys significantly underperformed compared to their stated wrought strength in literature^3,3^. Heat treating of wire-arc DED IN718 has been shown to improve the tensile properties, Seow et al. reported a room-temperature (RT) tensile performance of 86% of the wrought UTS with a modified heat-treatment^4^.

Bhujangrao et el. investigated the high temperature performance of WAAM IN718 in comparison with the wrought material and found that the formation of Laves phases lead to a reduction in performance in WAAM material, which they say is due to the brittle behaviour of Laves phase which act as a preferred fracture path^5^. The work of Lan et al. also reports the formation of Laves phase amongst dendritic arms and its association with cracking^8^. Artaza et al. investigated methods of controlling crack formation in WAAM IN718, in the study they found that using an interpass cooling strategy controls the formation of Laves phases and reduces crack formation^6^.

In using in-situ rolling with laser DED, Li et al. found that through the use of mechanical rolling of deposited layers, Laves phases formed in IN718 were more dispersed and found in a lower volume fraction compared with as deposited material. They also found that rolling assisted laser DED enhanced the tensile properties of IN718^9^.

To understand in more detail the effect the WAAM process has on the high temperature tensile properties of AD and heat-treated IN718 for a high-speed flight application, testing was conducted from RT—1000 °C. The application is expected to subject external structures to service temperatures as high as 1000 K (727 °C) and 1200 + K (927 °C) for components in the propulsion flow path.

## Method

**Figure 1:**
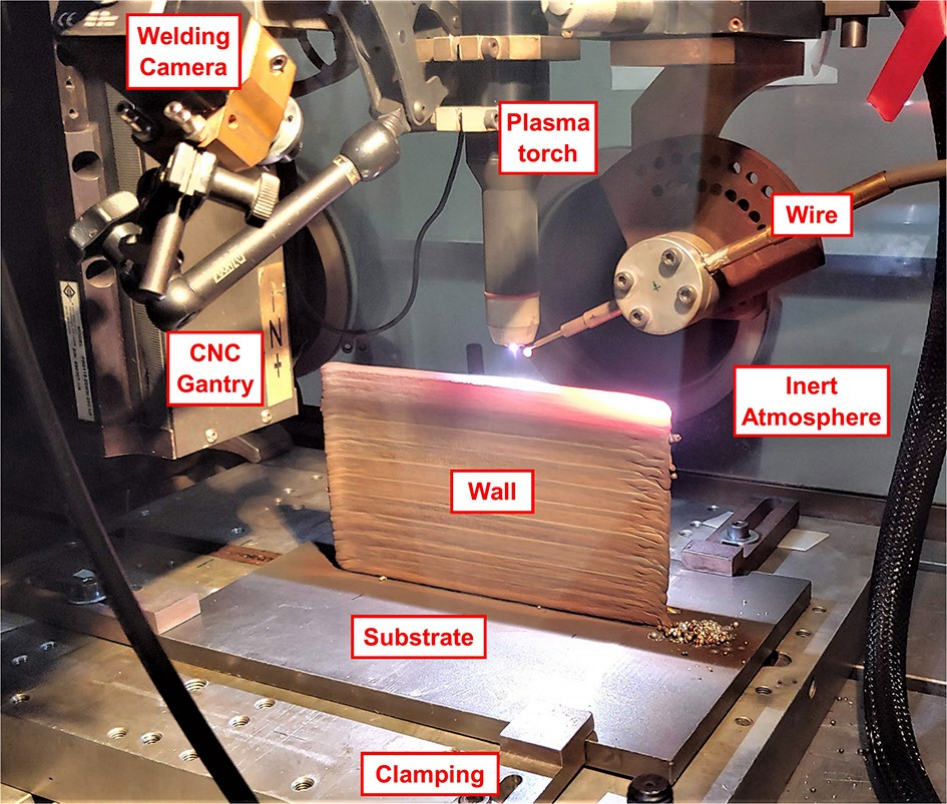
Experimental WAAM set-up^7^.

A wire-arc directed energy deposition (DED) process, commonly known as wire + arc additive manufacturing (WAAM) was used to deposit a 1.2 mm diameter Inconel 718 (IN718) wire. The experimental setup included: a three-axis linear CNC system, a Migatronic 320 A AC/DC plasma power source, a water-cooled plasma torch mounted to an adjustable jig on the CNC system, an external wire feeder, and a glove box housing an argon atmosphere, controlled using an oxygen analyser, at a level below 800 ppm of oxygen. The experimental set-up is shown in Fig. 1. The experimental set-up has been kept consistent with the previous work of James et al. on the WAAM deposition of the same alloy^7^.

Wall structures were deposited on to one side of the substrate in a single direction. The following parameters were used for the deposition: an arc current of 180 A, a wire feed speed of 1.8 m/min, a torch travel speed of 5 mm/sec, a torch to work distance of 8 mm, and finally an inter-pass temperature of 170 °C after approx. 3 min of cooling.

After deposition, samples for testing were extracted from the built WAAM wall and manufacturing into tensile specimens. Prior to testing, half of the specimens underwent an age hardening heat-treatment conforming to the industry standard applied on wrought alloys. Specimens underwent a process consisting of solutionising for one hour at 970 °C, followed by water quenching, then a further aging process for eight hours at 718 °C, after which samples were allowed to cool inside the furnace to 620 °C where they were held for a further eight hours. After completing the aging process samples were air quenched.

**Figure 2:**
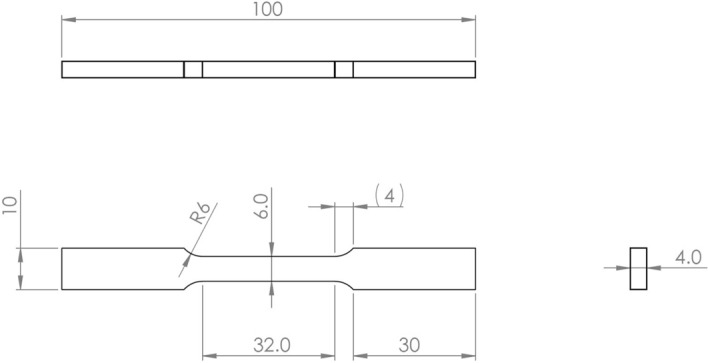
RT Tensile testing coupon conforming to ASTM E8M sub-sized specimen. X ± 0.5 mm, X.X ± 0.1 mm. (Not to scale).

**Figure 3:**
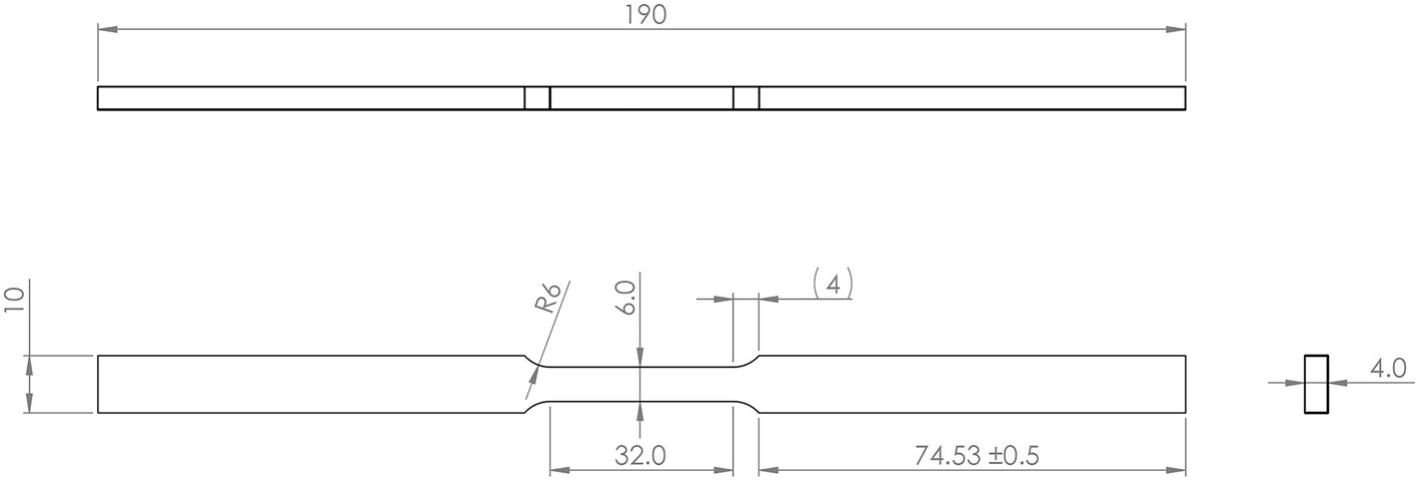
Elevated temperature tensile coupon used at 538–1000 °C, conforming to requirements of ASTM E21. X ± 0.5 mm, X.X ± 0.1 mm. (Not to scale).

To understand the performance of WAAM IN718 and the effect of post-deposition heat treatment, tensile specimens were tested at room temperature (RT), 538, 760 and 1000 °C, in both as-deposited (AD) and heat treated (HT) conditions. Tensile tests conformed to ASTM E8(M) for RT tests and E21 for high temperature testing. Drawings of the specimens used are given in Figs. 2 and 3. All testing was carried out using an Instron 8801 Servo-hydraulic Universal Testing System with a strain rate of 0.005 min^-1^ until the onset of plastic deformation and thereafter a crosshead speed of 1.6 mm/min. High temperature specimens were held for 30 min at the testing temperature, before testing commenced. Specimens were extracted from several locations on the WAAM wall to minimise variation in results due to any aging effect on the alloy from the WAAM process.

Specimens were also extracted for microstructural evaluation. Samples were extracted from the WAAM walls in build direction (BD)—through-thickness (TT) cross-sections, and were then prepared for metallographic analysis by mounting, grinding, and polishing successively. To reveal the microstructure, samples were swab etched for 10 s using Kalling’s reagent no. 2. Specimens were observed optically using a Leica DM 2700 M Microscope, and under scanning electron microscope (SEM) using a TESCAN Vega 3 SEM.

## Results and discussion

### Tensile performance

**Figure 4:**
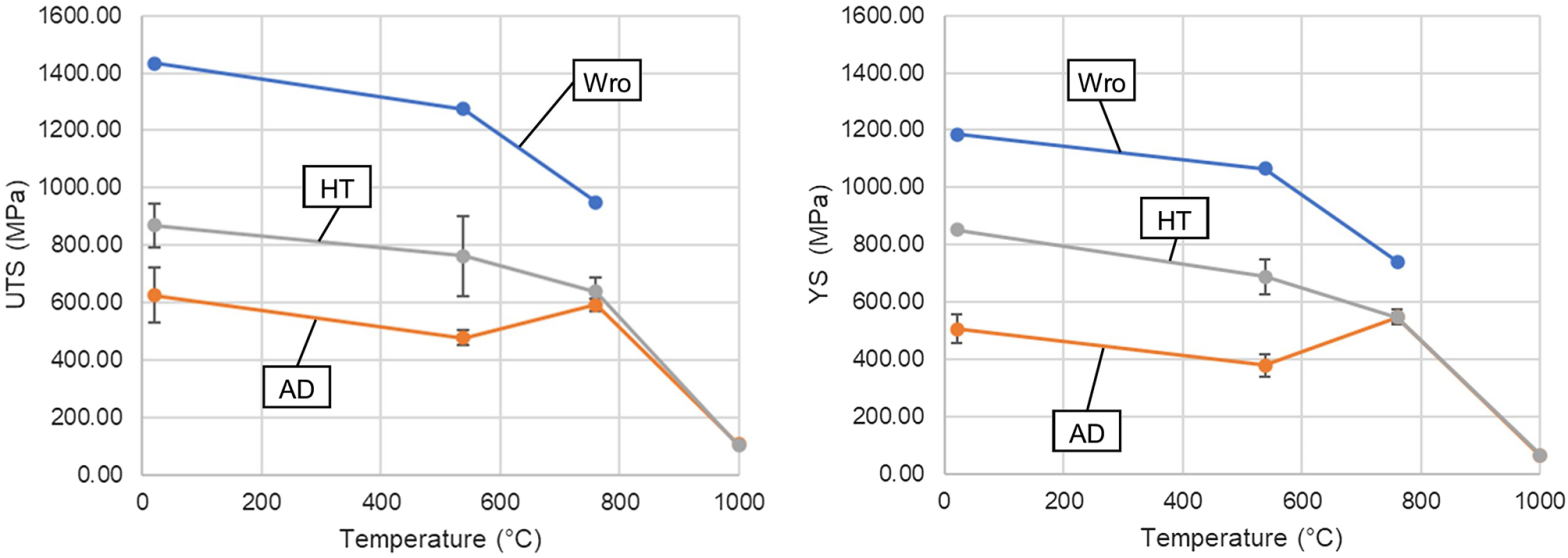
Graphical representation of data presented in Table 1. UTS (left), YS (right). (Colour in print).

The mechanical performance at the range of temperatures is given in both Table 1 and Fig. 4. Results are presented alongside the literature values for IN718 in its wrought condition. As expected from the literature data a decrease in performance was observed with increased testing temperature. The performance of the WAAM material lags behind the wrought condition.

**Table 1:** Tensile results comparing Wrought data (Wro)^12^ with AD and HT material from RT-1000 °C.

Temperature (°C)	UTS (MPa)			0.2% YS (MPa)			Elastic modulus E (GPa)		
	Wro	AD	HT	Wro	AD	HT	Wro	AD	HT
RT	1435	626	870	1185	505	852	200	125	178
538	1275	478	761	1065	378	687	171	153	152
760	950	592	639	740	546	547	154	125	124
1000		107	102		62	64		69	89

AD material achieves on average just 40% of the wrought strengths for RT and 538 °C tests, however when tested at 760 °C the average performance increases to 62% of wrought UTS and 74% of the YS. The increase in comparative performance at the 760 °C testing temperature indicates an in-test aging effect, where the material has been subjected to its aging temperature during testing. This is confirmed through the results of the HT specimens, which achieve a much more consistent comparative performance with the wrought data. HT specimens achieve 60% of the wrought UTS for RT and 538 °C and 67% at 760 °C.

When tested at 1000 °C the difference between AD and HT specimens is less apparent, and the difference in the elastic modulus is negligible throughout testing.

The elastic modulus, which is approx. 86% of the wrought material for both AD and HT specimens, this is thought to be due to minor changes in chemical composition that occur during heat treatment, as discussed by Parveen and Murthy^10^. This could be true for both AD and HT specimens due to the aging effect cause by the WAAM process, as seen previously by Xu et al.^11^.

### Microstructure

**Figure 5:**
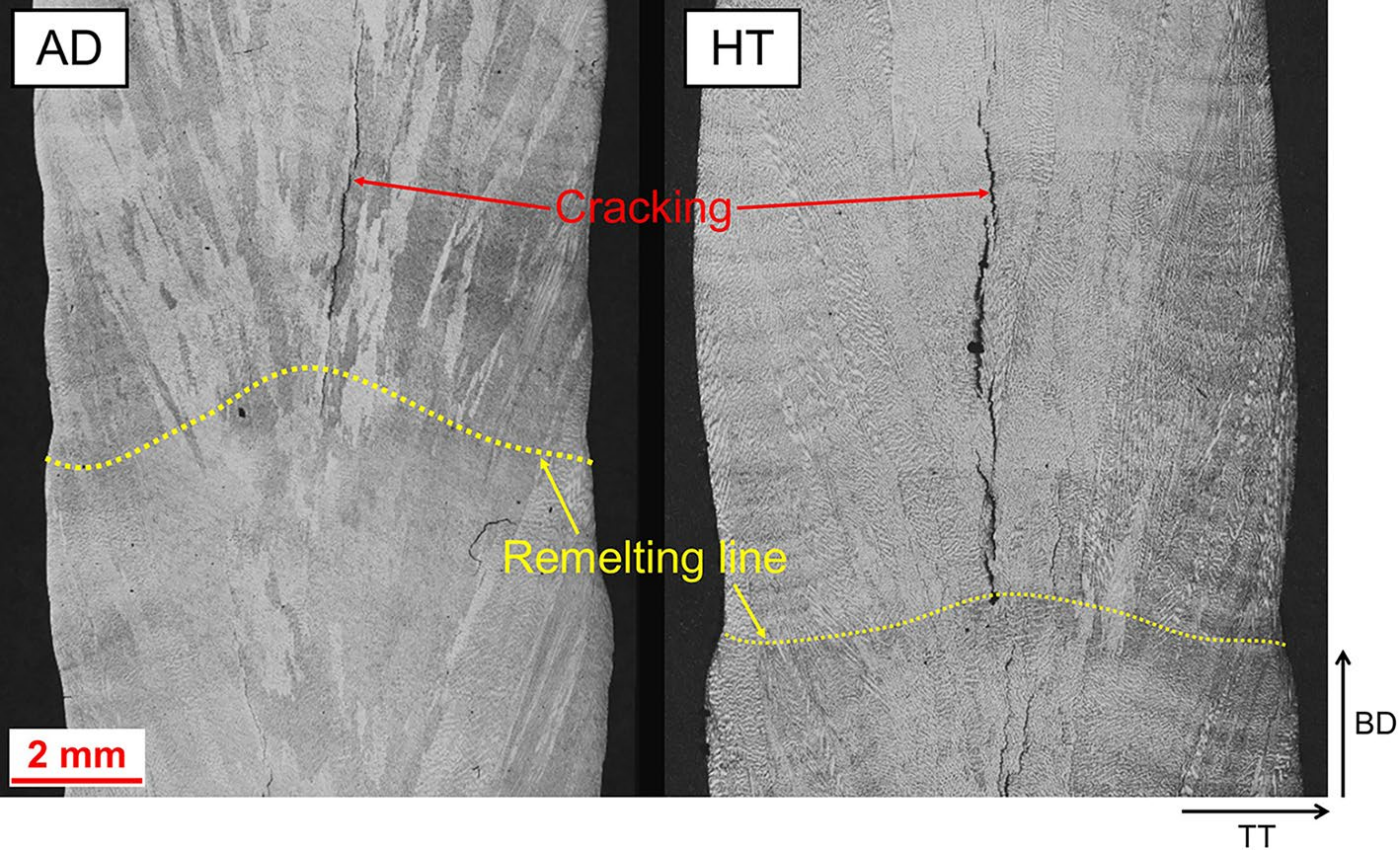
Overall microstructure.

The overall microstructure of AD and HT material is presented in Fig. 5. Significant amounts of cracking are observed throughout the build material, and cracking appears more severe after heat treatment.

**Figure 6:**
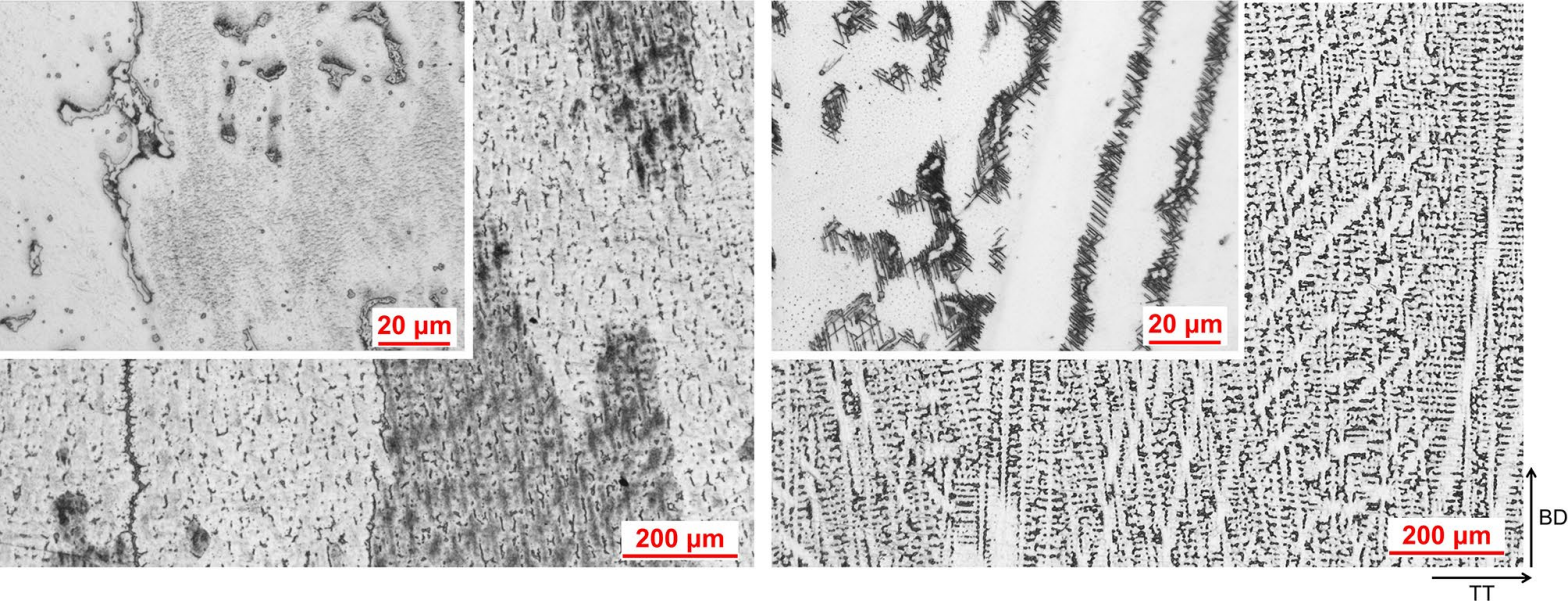
AD and HT microstructure. AD left, HT right.

**Figure 7:**
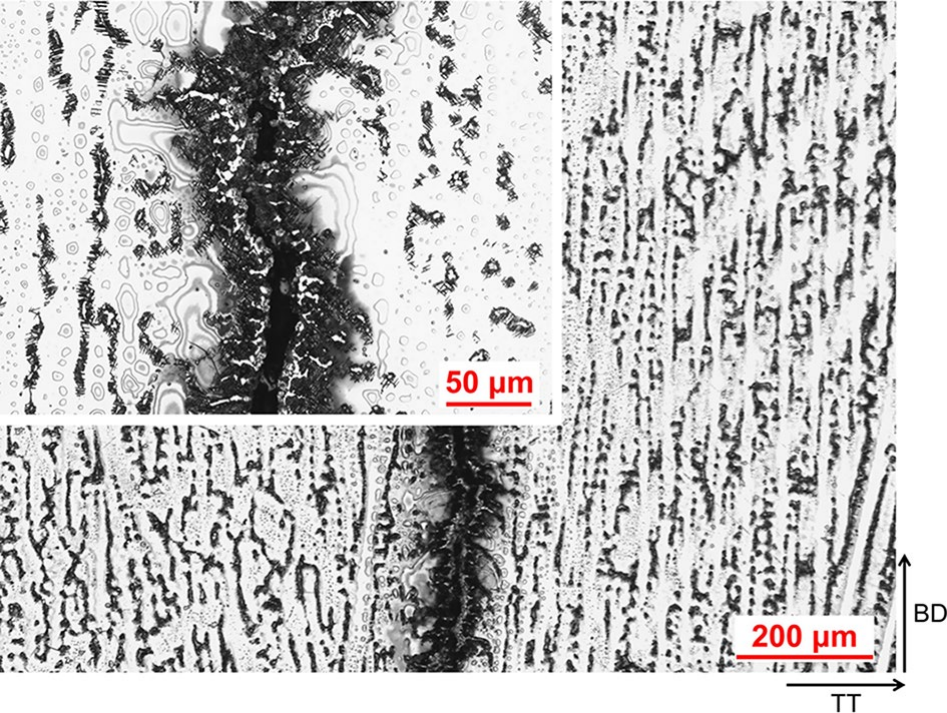
Secondary phases at crack edge in HT condition.

**Figure 8:**
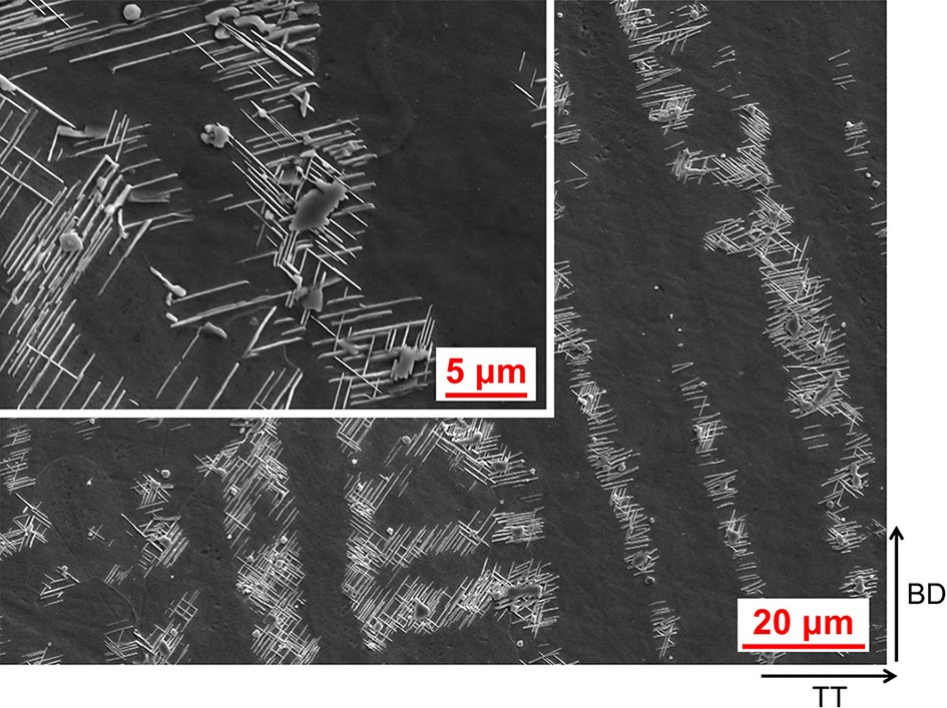
SEM view of secondary phases in HT condition.

A comparison between AD and HT microstructure is presented in Fig. 6. There is a great deal more precipitation visually observed in the HT condition compared to AD condition, which is not unexpected. In AD condition the microstructure exhibits chainlike islands of precipitates which are seen at the dendrites, and when the alloy has undergone heat-treatment these precipitates are seen to be surrounded by needle like precipitates in a Widmanstätten-Thomson pattern, which are understood to be Laves surrounded by an acicular δ phase, which was also reported by Xu et al. in IN718 produced by wire-arc DED^13^. A significant amount of cracking was also observed in both AD and HT condition, as seen in the micrographs. The crack edge appears to contain the same secondary phases observed throughout the matrix, and again when heat-treated precipitates an acicular phase locally (Fig. 7). The precipitation of these acicular phases can be seen in greater detail under SEM, presented in Fig. 8.

The formation of these phases at the crack edges suggests that the phases are detrimental to performance causing cracking during solidification, which is undoubtedly a contributing factor to the shortfall in performance observed against the wrought values.

The structure of IN718 produces using wire-arc DED, is known to be formed of large columnar grains, which are detrimental to the age hardened matrix. James et al. observed the wider microstructure of IN718 as well as other alloys in a previous study^3^.

**Figure 9:**
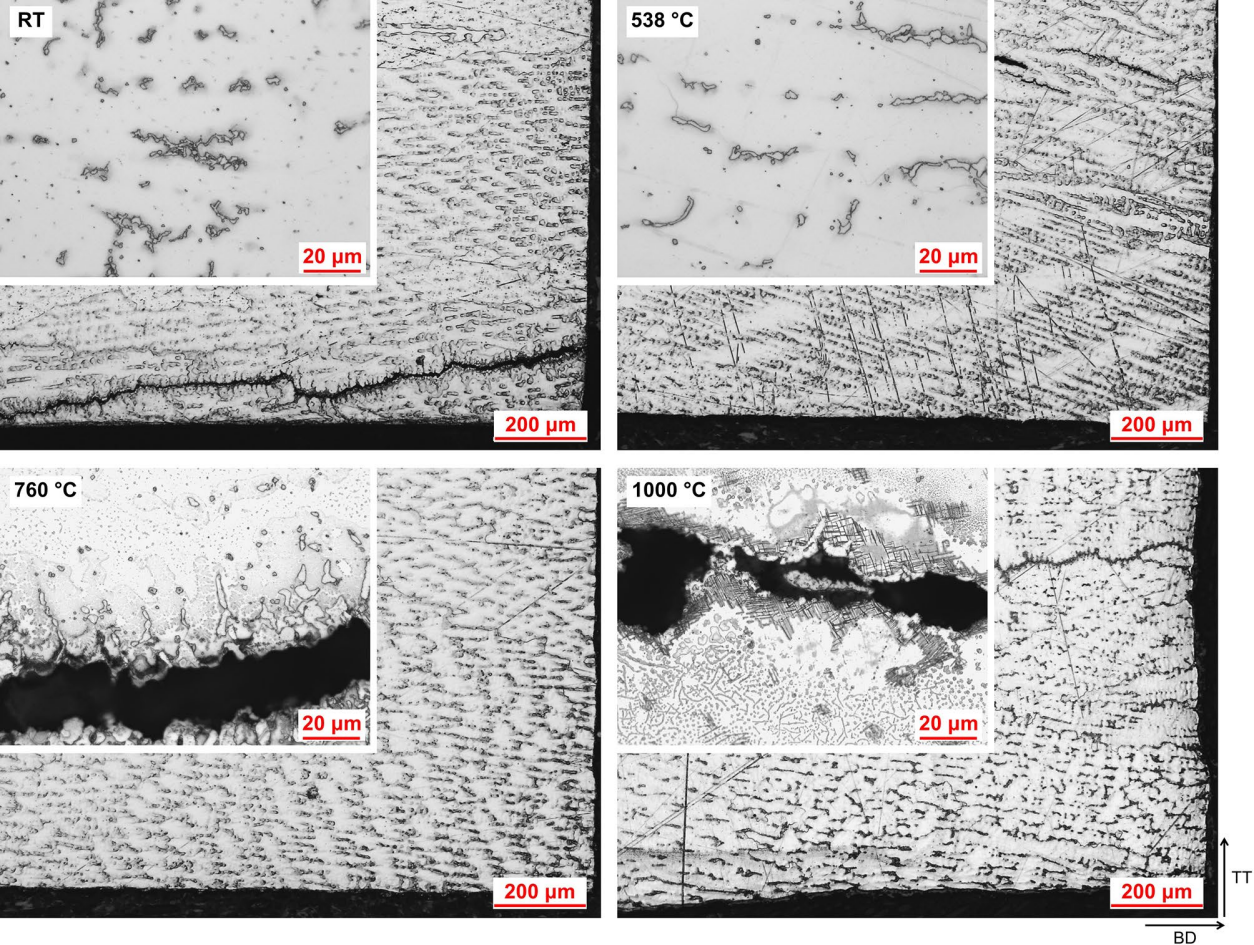
Microstructure behind fracture surface of AD tested specimens showing the aging effect of the testing temperatures.

As suggested earlier it is believed that testing at elevated temperature has caused the AD samples to age during testing, owing to an increase in the performance of AD samples at the 760 °C test, where AD samples meet the performance of the HT specimens. The microstructure behind the fracture surface of AD specimens is presented in Fig. 9 at each tested temperature. It can be clearly seen in the microstructure the precipitation of phases at 760 °C as well as the formation of acicular phases after testing at 1000 °C.

## Conclusion

1. Depositing IN718 using wire-arc DED impacts the tensile performance. AD and HT material achieves 40 and 60% of the wrought UTS respectively in the range of RT—538 °C.
2. Heat-treating AD material using the industry standard treatment increases the performance but does not result in an increase to wrought performance.
3. Testing AD material at 760 °C leads to an increase in performance owing to an in-test aging effect.
4. Wire-arc DED causes solidification cracking in IN718, where secondary phases are found at the crack edges.

## Data Availability

The datasets generated and analysed during the current study are not publicly available as they are the subject of an ongoing study, but are available from the corresponding author on reasonable request.
